# Inducing extra copies of the *Hsp70* gene in *Drosophila melanogaster* increases energetic demand

**DOI:** 10.1186/1471-2148-13-68

**Published:** 2013-03-19

**Authors:** Luke A Hoekstra, Kristi L Montooth

**Affiliations:** 1 Department of Biology, Indiana University, Bloomington, IN 47405, USA

**Keywords:** Copy number variation, Gene expression, Gene duplication, Hsp70, Heat-shock response, Adaptation, Drosophila

## Abstract

**Background:**

Mutations that increase gene expression are predicted to increase energy allocation to transcription, translation and protein function. Despite an appreciation that energetic tradeoffs may constrain adaptation, the energetic costs of increased gene expression are challenging to quantify and thus easily ignored when modeling the evolution of gene expression, particularly for multicellular organisms. Here we use the well-characterized, inducible heat-shock response to test whether expressing additional copies of the *Hsp70* gene increases energetic demand in *Drosophila melanogaster*.

**Results:**

We measured metabolic rates of larvae with different copy numbers of the *Hsp70* gene to quantify energy expenditure before, during, and after exposure to 36°C, a temperature known to induce robust expression of *Hsp70*. We observed a rise in metabolic rate within the first 30 minutes of 36°C exposure above and beyond the increase in routine metabolic rate at 36°C. The magnitude of this increase in metabolic rate was positively correlated with *Hsp70* gene copy number and reflected an increase as great as 35% of the 22°C metabolic rate. Gene copy number also affected *Hsp70* mRNA levels as early as 15 minutes after larvae were placed at 36°C, demonstrating that gene copy number affects transcript abundance on the same timescale as the metabolic effects that we observed. Inducing *Hsp70* also had lasting physiological costs, as larvae had significantly depressed metabolic rate when returned to 22°C after induction.

**Conclusions:**

Our results demonstrate both immediate and persistent energetic consequences of gene copy number in a multicellular organism. We discuss these consequences in the context of existing literature on the pleiotropic effects of variation in *Hsp70* copy number, and argue that the increased energetic demand of expressing extra copies of *Hsp70* may contribute to known tradeoffs in physiological performance of extra-copy larvae. Physiological costs of mutations that greatly increase gene expression, such as these, may constrain their utility for adaptive evolution.

## Background

Cellular respiration produces adenosine triphosphate (ATP), the energy currency of the cell. The generation and maintenance of traits that contribute to organismal fitness often requires energy consumption, and the metabolic rate of an individual changes dynamically to meet these requirements [1,2]. Fluctuating biotic, abiotic and cellular environments challenge metabolic homeostasis; and yet, in the face of this, many organisms have evolved physiological responses that require energy investment to routinely resist environmental stress [3].

One of the best-characterized physiological responses, the cellular stress response, involves the massive up-regulation of molecular chaperones (e.g. [4,5]). This response is induced at sub-lethal stress exposures and confers enhanced survival when organisms encounter potentially lethal exposures later in life (i.e. inducible tolerance). In *Drosophila melanogaster*, more genomic copies of the inducible molecular chaperone *Hsp70* increases inducible thermotolerance [6], and variation in *Hsp70* expression explains evolved differences in inducible thermotolerance among both laboratory and natural isolates [6,7]. Gene duplication of the *Hsp70* locus is responsible for divergent evolution of this response among *Drosophila* species, and the subsequent conservation and retention of extra genomic copies of *Hsp70* within *Drosophila melanogaster* is taken as evidence for adaptive evolution of increased stress tolerance via increased *Hsp70* inducible expression [8-10].

Mutations that increase gene expression, such as the duplication of an *Hsp70* gene, should increase energy demand, due to the ATP-dependent processes of gene expression, protein production, and protein function (e.g. [11]). In fact, the energetic costs accompanying increases in gene expression may significantly limit the retention of duplicates in the genomes of unicellular organisms [12-14] and constrain horizontal gene transfer [15]. Whether gene expression places significant demands on the energy budgets of multicellular organisms is less explored [12,16], but the widespread observation of weak selection on codon and amino acid usage suggests that even small differences in the efficiency of molecular processes can influence fitness and therefore have consequences for molecular evolution [17,18]. In order for mutations that increase gene expression to contribute to adaptive evolution, the fitness advantages of increased gene expression must outweigh any decrease in fitness associated with the energetic costs of increased gene expression.

The inducible stress response in *D. melanogaster* cells represents a remarkable reallocation of molecular machinery and cellular resources away from global transcription and translation of the genome and towards rapid induction of the multi-copy *Hsp70* locus, resulting in high levels of this ATP-dependent molecular chaperone [19-23]. The full organismal benefits of this inducible tolerance, however, are delayed 1-8 hours after transcript and protein begin to accumulate, depending on lifestage. These observations suggest that there may be transient, but significant, energetic costs of inducing this locus, and we predicted that these costs would increase as a function of increased *Hsp70* gene copy number. Here we use dynamic and sensitive measures of larval metabolic rate during the heat shock response to demonstrate that increased gene copy number increases the energetic cost of this inducible response in *D. melanogaster*. We hypothesize that this increase in energy expenditure represents replenishing of the ATP pool in response to the cost of rapid, increased gene expression and protein accumulation (sensu [24-28]). We discuss these results in the context of known pleiotropic effects of the *Hsp70* locus, and we argue that the energetic costs associated with increased gene expression may sets upper bounds on the persistence of genetic material available for adaptive evolution.

## Methods

### Drosophila genotypes and maintenance

Larvae from the genotypes *Hsp70*^*A-Ba-*^*, Hsp70*^*CisIII*^*,* and *Hsp70*^*TraIII*^, containing 3, 6 and 12 copies of *Hsp70*, respectively [29], were reared at controlled densities on standard cornmeal-agar food at 22°C (12 h:12 h L:D). All three genotypes were engineered from the same 6-copy *Hsp70* genetic background (*w*^1118^). Reduction in gene copy number (*Hsp70*^*A-Ba-*^) was achieved through homologous recombination that removed 3 copies of *Hsp70*[30]. We verified the presence of this deletion using a PCR assay according to [29]. The extra copy genotype (*Hsp70*^*TraIII*^) results from a transgene insertion of 6 additional copies on the third chromosome, and the 6-copy line (*Hsp70*^*CisIII*^) is the precise control for this insertion, differing only in the absence of the extra copies [31]. The presence of this insertion was confirmed by eye color, as the transgene insertion contains a wildtype allele of *white* that restores red eye color in the white-eyed *w*^1118^ genetic background. The insertion and deletion events are not known to disrupt other loci and the remainder of the genome is shared among lines; thus, differences among genotypes can be attributed solely to the effects of differences in copy number. For *Hsp70*^*A-Ba-*^*, Hsp70*^*CisIII*^*,* and *Hsp70*^*TraIII*^, variation in the expression of other heat-shock protein genes is small or negligible in response to the induction protocols we used [29].

### Larval metabolic rate

We sought to quantify whether larval metabolic rate, as a measure of energetic expenditure, changed dynamically upon induction of the *Hsp70* locus and whether this response differed among *Hsp70* gene copy number genotypes. The volume of CO_2_ (${\dot{V}\mathrm{CO}}_{2}$) produced by *D. melanogaster* larvae is a good measure of aerobic metabolic rate, and flow-through respirometry allows dynamic, high-resolution quantification of metabolic CO_2_ production [32]. We used this technique to quantify metabolic rate of *D. melanogaster* larvae with different *Hsp70* gene copy numbers before, during, and after induction of the heat-shock response at 36°C. 60 min at 36°C is a sub-lethal exposure that has been documented to rapidly induce *Hsp70* expression and Hsp70 protein accumulation [6,21,29]. Replicate pools of five, pre-wandering, third-instar larvae of the same genotype were placed on a tared, 0.5 mL food pellet, massed to the nearest μg, and then placed into a glass flow-through respirometry chamber (RC-M, Sable Systems International, Inc., Las Vegas, NV). Each respirometry experiment resulted in a respirometry tracing of metabolic rate from a pool of larvae that experienced the following series of treatments: 15 min at 22°C, 60 min at 36°C, and 100 min at 22°C. Survival of these larvae to adult eclosion was high and did not differ among genotypes (Additional file 1: Figure S1).

**Figure 1:**
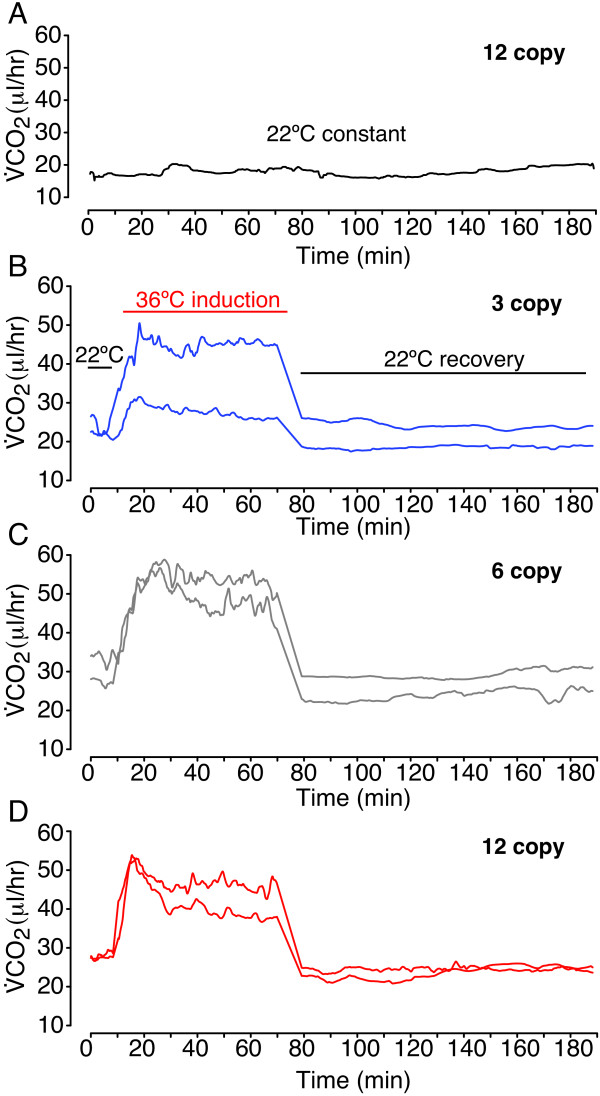
**Representative metabolic rate (**$\dot{V}CO_{2}$**) tracings from larvae with different *Hsp70 *gene copy numbers before, during, and after 36°C exposure.** Metabolic rate was measured as the volume of CO_2_ (μL hr^-1^) produced by pools of five larvae before, during and after exposure to the *Hsp70*-inducible temperature of 36°C. **A**. Larvae left at 22°C never had a rise in metabolic rate, as observed at 36°C. **B**, **C** and **D**. The increase in metabolic rate due to the *Q*_10_ effect of increased temperature is readily apparent when 3-copy (**B**), 6-copy (**C**) and 12-copy (**D**) larvae are moved from 22°C to 36°C. All genotypes exhibited a transient increase in metabolic rate above the 36°C RMR that always occurred within the first 30 minutes of 36°C exposure. The metabolic rate of larvae with 12 copies of *Hsp70* rises above and beyond that of other genotypes. Shown are tracings representative of the average and maximum rise in metabolic rate observed for each genotype. Different placement of tracings on the y-axis represents differences in overall metabolic rate due to mass effects, but mass did not differ among genotypes (*F*=2.66, *P*=0.078), nor did the 22°C or 36°C RMRs (Table 1 and Additional file 2: Table S1). Variation in the *Q*_10_ among different samples of larvae is also apparent; however, *Q*_10_ also did not differ among genotypes (*F*=0.76, *P*=0.384). Tracings begin five minutes after larvae are placed at 22°C.

Metabolic rates were measured for 23 independent pools of larvae per genotype as the amount of CO_2_ produced in a CO_2_-free, hydrated air stream (flow rate = 70 ml min^-1^). Water vapor was selectively removed prior to measuring the CO_2_ produced by the larvae, which was sampled once per second using an infrared CO_2_ gas analyzer (LI-COR, LI-7000, Lincoln, Nebraska). Before and after each of the three temperature phases of each experiment, baseline recordings from empty respirometry chambers were used to correct for any drift in the CO_2_ measurement during each phase using the two-endpoint automatic method in the software package Expedata (Sable Systems). These baseline-corrected measures of CO_2_ were then exported to the statistical package R, version 2.11.0 for further analyses [33]. Baseline-corrected CO_2_-values were converted from p.p.m. to μl hr^-1^ using the flow rate of 70 ml min^-1^. Figure 1 shows representative respirometry tracings of these ${\dot{V}\mathrm{CO}}_{2}$ data for each genotype, smoothed with a running mean function as described below.

For each sample of larvae, we estimated routine metabolic rate (RMR) as the mean ${\dot{V}\;\mathrm{CO}}_{2}$ over a stable 10 min period during the initial 22°C exposure (22°C RMR) and as the lowest 10 min mean during the 36°C induction (36°C RMR). For statistical analyses, measures of RMR were mass-corrected by using the residuals from a regression of RMR on body mass after a comparison of slopes tests supported a common slope for all genotypes. However, since there were no significant genotype-by-mass interactions or effects of genotype on mass, mass-correction did not qualitatively change the results of our analyses (Additional file 2: Table S1). Regressions of log-transformed data did not significantly improve the fit of the models, because the relationship between metabolic rate and mass is linear across the small range of masses in our experiment. The values of RMR in Table 1 report the mean residuals for each genotype from the regression of ${\dot{V}\mathrm{CO}}_{2}$ on body mass, with the grand mean of the fitted values from each regression added back to all values to provide meaningful units and scale. These values are the mass-corrected measure of RMR for 5 larvae (μl hr^-1^).

**Table 1:** **Effects of *Hsp70 *copy number genotype on metabolic rate**

	***Hsp70 *copy number genotype**		
**Measure of metabolic rate**	**3 copy**	**6 copy**	**12 copy**
Mass-corrected 22°C RMR^1^	25.03 ± 0.6 (a)^2^	26.37 ± 0.5 (a)	26.47 ± 0.8 (a)
Mass-corrected 36°C RMR	39.94 ± 0.6 (a)	41.65 ± 0.9 (a)	39.57 ± 1.1 (a)
Mass-corrected 36°C maxMR	45.10 ± 0.7 (a)	48.35 ± 1.1 (b)	49.16 ± 1.2 (b)
*Q*_10_^3^	1.41 ± 0.024 (a)	1.38 ± 0.024 (a)	1.34 ± 0.029 (a)
Rise in 36°C MR	5.15 ± 0.3 (a)	6.69 ± 0.7 (a)	9.20 ± 0.7 (b)
Rise as a % of 22°C RMR	20.7 ± 1.2 (a)	25.4 ± 2.7 (a)	34.7 ± 2.5 (b)
Rise as a % of 36°C RMR	13.0 ± 0.8 (a)	16.4 ± 2.0 (a)	23.5 ± 2.0 (b)
% Recovery of 22°C RMR, 0-20 min^4^	92.6 ± 2.1 (a)	88.9 ± 2.4 (a)	86.7 ± 2.5 (a)
% Recovery of 22°C RMR, 95-115 min^4^	95.6 ± 2.1 (a)	93.5 ± 2.5 (a)	89.9 ± 2.5 (a)

^1^ Units for all measures other than percentages are μL CO_2_ hr^-1^ ± S.E. for 5 larvae. See methods for description of mass correction.

^2^ Within each trait, different letters indicate significant differences between copy number genotypes (*P*_*Tukey’s*_*<*0.05).

^3^Q10=36°CRMR22°CRMR1036−22.

^4^ Recovery was calculated from the mean metabolic rate during the first 20 min following return to 22°C and the final 20 minutes of the respirometry experiments as described in the methods.

Temperature increases metabolic rate due to increased thermodynamics, and we quantified this increase in RMR as Q10=36°CRMR22°CRMR1036−22. In the majority of respiratory experiments, we observed a transient increase in metabolic rate above and beyond the *Q*_10_ that always occurred in the first half of the 36°C exposure. We summarized this rise in metabolic rate as the difference between the maximum ${\dot{V}\mathrm{CO}}_{2}$ value (maxMR) and the 36°C RMR. The maxMR was calculated from data smoothed with a running mean function to ensure that the maximum reflects a true and sustained peak rather than any high-frequency noise or random fluctuation in the data. All qualitative effects of genotype were robust to the choice of averaging window. We also calculated the percent recovery of the 22°C RMR when larvae returned to 22°C after 36°C exposure. We calculated this value as either the mean MR in the first 20 minutes or in the last 20 minutes of the recovery phase of the experiment divided by the initial 22°C RMR for each pool of larvae. These summary statistics of metabolic rate (*Q*_10_, rise, and recovery) compare metabolic rates of the same pool of larvae at two points in the respirometry experiment. We calculate these relative metabolic rate measures using non-mass corrected data, given that we would use the same mass data to correct both measures of metabolic rate. We then confirmed that these relative metabolic rate measures were independent of mass by testing for a relationship between the measure and mass (*P*>0.25 for all measures). Genotype effects for all metabolic rate measures were tested using ANOVA and Tukey’s post-hoc contrasts in the statistical package R, version 2.11.0 [33].

### Larval activity at 36°C

The necessity of providing a food source, and the small size of *D. melanogaster* larvae, prevented the quantification of larval activity during respirometry. However, we wanted to determine whether genotype differences in the magnitude of the rise in metabolic rate during 36°C were caused by genotype differences in activity. Therefore, we independently assessed the effect of 36°C exposure on larval activity using blind, randomized observational trials. Five larvae from a randomly chosen genotype were placed onto a thin, 0.5mL food pellet inside the well of a glass depression slide. This glass slide was placed on top of a thermal plate at either 22°C or 36°C. A 4 mm × 4 mm grid printed onto transparency film was loosely placed over the top of the slide for scoring larval position and movement. In 60-second bouts, a focal larva was identified and activity was scored as the number of grid-squares passed through within the 60-second viewing period. Genotype effects were tested using ANOVA.

### Larval gene expression during 36°C exposure

Measures of *Hsp70* induction in larvae of these copy number genotypes after a 60 min exposure to 36°C have been reported previously [29]. However, given that we observed an effect of *Hsp70* copy number on metabolic rate in the first 0-30 min after exposure to 36°C, we tested for gene copy number effects on *Hsp70* induction prior to 60 min of 36°C exposure. To do this, we used SYBR-green fluorescence based quantitative real-time PCR (qRT-PCR) assays to quantify *Hsp70* mRNA abundance in larvae exposed to 36°C for 0, 15, 30 and 60 minutes, relative to the commonly used reference gene *Actin5C* (*Act5C*) [34,35]. *Actin* message is known to be stable in cells during 36°C exposure [20]. qRT-PCR assays were run on a Stratagene Mx3005P (Agilent Technologies).

Detailed methods for the quantification of *Hsp70* gene expression in larvae of these genotypes have been described previously [29]. Briefly, replicate groups of 20 third-instar larvae from each genotype were placed into 1.5 mL microcentrifuge tubes with 0.5mL of food. The larvae were then heated in a heat block for 0, 15, 30, or 60 min at 36°C. This method of heating closely matched the shift to 36°C that larvae experienced in the respirometry chambers. After the given treatment time, the 20 larvae were placed into a new tube, frozen in liquid nitrogen and stored at -80°C. We extracted RNA using a Trizol-based method and synthesized cDNA using oligo-dT primers following protocols in [29]. Four independent replicate cDNA samples per genotype and treatment were used for qRT-PCR, and at least four technical replicate measures were used to estimate mean *Hsp70* and *Act5C* abundance (Ct) per sample using standard SYBR-green qRT-PCR protocols. The raw measure of mRNA abundance is the Ct, which is the PCR cycle at which the fluorescence detected from gene amplification exceeds a set threshold. A more abundant sample will have a lower Ct, while a less abundant sample will have a higher Ct. *Hsp70* primers were designed to detect expression from all *Hsp70* gene copies. Standard curves using 2- to 32-fold dilution series of cDNA from either 3-copy larvae exposed to 0 min at 36°C (our least abundant samples) or 12-copy larvae exposed to 60 min at 36°C (our most abundant samples) were used to optimize the assay, and the slopes from these curves were used to determine the efficiencies of the assays (*E* = 10^− 1/*slope*^). Standard curves were linear across this range of Ct values, confirming the ability of our assay to detect changes across our most and least abundant samples. A qRT-PCR assay with perfect efficiency (*E=*2) will detect a 2-fold difference in mRNA abundance as a difference of one Ct (i.e. Ct measures gene expression on a log2 scale), and our assays had high efficiencies of 1.87 and 1.95 for *Hsp70* and *Act5C*, respectively.

Data were pre-processed in MxPro v4.10 Build389 Schema85 to obtain the Ct measures from the qRT-PCR amplification curves. Statistical analyses of genotype effects within time points and calculations of relative fold changes across time points were performed using R, version 2.11.0 [33]. We used an analysis of covariance (ANCOVA) to test for differences in *Hsp70* Ct values among genotypes within time points, controlling for the reference gene by including *Act5C* Ct as a covariate for each sample in the analysis. This analysis avoids dividing by the reference gene Ct and does not assume that the reference and target gene assays have the same efficiencies. There was no effect of treatment, genotype or their interaction on *Act5C* expression (*P*>0.09 for all factors), and the slope of the relationship between *Hsp70* and *Act5C* was similar across time points, with no evidence for genotype-specific slopes (*P>*0.12 at each time point). This suggests that *Act5C* is a good reference gene in this experiment, controlling for sample-to-sample variation in total mRNA extracted. Another standard approach is to analyze the *ΔCt* = *Ct*(*target*) − *Ct*(*reference*) for each sample. This analysis assumes equal efficiencies of target and reference gene assays. ANOVA of genotype effects on *Hsp70* ΔCt values within each time point yielded the same qualitative results as the ANCOVA approach (Additional file 3: Table S2).

We used two measures to calculate the relative fold change in *Hsp70* mRNA for each genotype across time points during exposure to 36°C, controlling for any change in the reference gene. First, we used the ΔΔCt method that assumes both assays have perfect efficiency and calculates relative fold change as 2^*ΔCt*(*sample*) − *ΔCt*(*control*)^, where the sample is from larvae exposed to either 15, 30 or 60 min at 36°C and the control value is from control larvae not exposed to 36°C. Each ΔCt in this equation is calculated as described above and accounts for expression of the reference gene. Second, we used the empirically determined assay efficiencies to calculate the fold change in *Hsp70* relative to the fold change in *Act5C* between the control larvae and the 36°C sample larvae at each time point as $\frac{{\left(E_{\mathrm{Hsp}70}\right)}^{\mathrm{\Delta C}t_{\mathrm{Hsp}70}\left(\mathrm{control}-\mathrm{sample}\right)}}{{\left(E_{\mathrm{Act}5C}\right)}^{\mathrm{\Delta C}t_{\mathrm{Act}5C}\left(\mathrm{control}-\mathrm{sample}\right)}}\;$[36]. Both calculations yield the same qualitative patterns (Additional file 4: Table S3), and we report in Table 2 the more conservative estimates that incorporate our assay efficiencies.

**Table 2:** **ANCOVA of gene copy number effects on relative *Hsp70 *mRNA abundance**

	**Duration of exposure to 36°C**			
**Factor**	**0 min**	**15 min**	**30 min**	**60 min**
*Act5C*^1^	*b* = 0.67 ± 0.13 ***	*b* = 0.98 ± 0.07 ***	*b* = 0.85 ± 0.07 ***	*b* = 0.64 ± 0.10 ***
Genotype	*F =* 1.34	*F =* 22.2 ***	*F =* 35.4 ***	*F =* 31.9 ***
Genotype effects^2^:				
6 copy – 3 copy	−0.66	−2.23 **	−1.56 ***	−1.76 ***
12 copy – 3 copy	−0.99	−1.90 **	−1.66 ***	−1.91 ***
12 copy – 6 copy	−0.33	+0.33	−0.09	−0.14

^1^ Reference gene expression included as a covariate; *b* is the slope estimate.

^2^ Estimates using Tukey’s post-hoc contrasts; smaller values of the Ct indicate higher expression levels.

* *P*<0.05, ** *P*<0.01, *** *P*<0.001.

## Results

### Hsp70 gene copy number affects metabolic rate during 36°C exposure

To quantify the effects of *Hsp70* induction on whole-organism energetics, we used flow-through respirometry to continuously measure larval metabolic rate via CO_2_ production before, during, and after a 60 min exposure to the non-lethal temperature of 36°C (Figure 1). This exposure induces a robust *Hsp70* response, during which larvae of these genotypes accumulate significant amounts of both *Hsp70* mRNA [29] and Hsp70 protein [6]. For the larvae we measured, the percent survival-to-adult eclosion after respirometry experiments exceeded 90% for all three copy number genotypes (Additional file 1: Figure S1). In insects like *Drosophila* that primarily metabolize carbohydrates via aerobic respiration to generate ATP, the production of CO_2_ is a good measure of energy expenditure [32]. Mass-corrected RMRs did not differ among genotypes at either 22°C (*F*=1.72, *P*=0.19) or 36°C (*F*=1.15, *P*=0.32), nor did the response of RMR to temperature (the *Q*_10_) differ among genotypes (*F*=1.54, *P*=0.22) (Table 1). This latter observation indicates that *Hsp70* copy number genotype does not perturb the overall thermodynamic effect of reaction rates on metabolic rate. The *Q*_10_ effect is readily apparent in the respirometry tracings as a large increase in ${\dot{V}\mathrm{CO}}_{2}$ when larvae were moved from 22°C to 36°C (Figure 1B-D).

**Figure 2:**
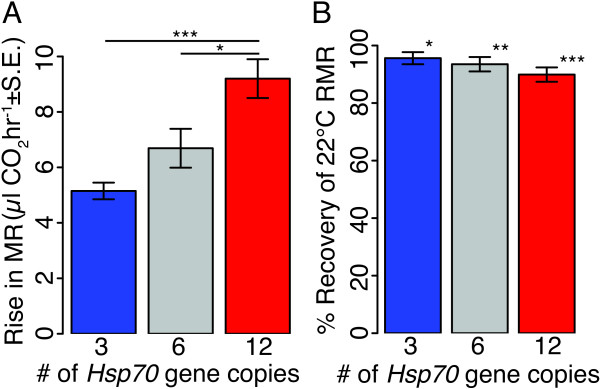
**Effect of *Hsp70 *copy number genotype on metabolic rate during and after induction of *Hsp70*. A**. The rise in metabolic rate during 36°C exposure (Rise in MR), calculated as the difference between the maxMR and the 36°C RMR, differs significantly among *Hsp70* genotypes (*F*=11.7*, P*<0.0001). The rise in metabolic rate of larvae with 12 copies of *Hsp70* is significantly greater than that of larvae with 3 copies (*P*_*Tukey*_=0.00003) or 6 copies of *Hsp70* (*P*_*Tukey*_=0.013) (*N*=23 replicate pools of larvae per genotype). **B**. After exposure to 36°C, larvae from all genotypes returned to a 22°C RMR that was lower than their initial 22°C RMR, and they failed to fully recover their initial 22°C RMR for the duration of the experiment. For each pool of larvae, we calculated the % Recovery of 22°C RMR as the mean metabolic rate 95-115 minutes after returning to 22°C divided by the initial 22°C RMR (prior to 36°C exposure). Plotted are the means of this % recovery statistic (± SE) for each genotype. Asterisks indicate that the difference in metabolic rate before and after 36°C exposure is significantly different from zero (Paired t-test, *P<0.05, **P<0.01, ***P<0.001).

Upon exposure to 36°C, we detected a rapid and transient increase in larval MR above and beyond the 36°C RMR (Table 1, Figures 1 and 2A). This resulted in a maximum metabolic rate that always occurred during the first half of the 36°C phase of the respirometry experiment, while estimates of the 36°C RMR always occurred in the latter half of the 36°C exposure. Control recordings of metabolic rate at 22°C never showed the type of dynamic rise in metabolic rate over and above the RMR seen at 36°C (e.g. Figure 1A). We quantified this increase in metabolic rate above the 36°C RMR as the difference between the smoothed maximum metabolic rate (maxMR) and the 36°C RMR. *Hsp70* copy number genotype has a significant effect on this rise in metabolic rate (*F*=11.66*, P<*0.00005; Table 1 and Figure 2A). Larvae with 12 copies of *Hsp70* experience a significantly larger rise in metabolic rate relative to larvae with either 3 copies (*P*_*Tukey*_=0.00003) or 6 copies (*P*_*Tukey*_=0.013; Figure 2A).

The magnitude of this metabolic rise is large relative to the RMR, reflecting an increase in MR that is 13-24% of the 36°C RMR and 21-35% of the 22°C RMR, depending on copy number genotype (Table 1). We observed that this rise in MR above and beyond the RMR can persist for a duration of 15 minutes, and that the magnitude of the rise increases 1.3-fold with a doubling of copy number from 3 to 6, and 1.4-fold with a doubling of copy number from 6 to 12. Finally, this rise in metabolic rate was not simply a consequence of increased activity during induction, as activity at 36°C was not significantly different among genotypes (*F*=1.49*, P*=0.22).

### Extra Hsp70 genes increase induction of Hsp70 as early as 15 minutes upon 36°C exposure

Given how soon the genotypic effects on the rise in metabolic rate occur after larvae are placed at 36°C, we tested whether gene copy number affected the dynamics of *Hsp70* induction as early as 15-30 minutes at 36°C. We measured relative *Hsp70* mRNA abundance in larvae of each of the copy number genotypes after 0, 15, 30 and 60 minutes at 36°C. As early as 15-30 minutes after being placed at 36°C, larval *Hsp70* mRNA abundance was significantly greater in 6- and 12-copy genotypes relative to 3-copy genotypes (Table 2), a time that is consistent with the genotype effects on the rise in metabolic rate that we observed. We find that, in all genotypes, the greatest change in *Hsp70* mRNA abundance occurs in the first 30 minutes of 36°C exposure (Table 3). After 30 minutes at 36°C, larvae with 12, 6 and 3 gene copies increase *Hsp70* mRNA abundance to 583, 428 and 277 times their basal levels, respectively (Table 3). Thus, a doubling of gene copy number does not double the rate of *Hsp70* mRNA accumulation, consistent with the self-limiting regulation of this locus [19,29,37]. The 12-copy genotype had the greatest fold induction of *Hsp70* mRNA and the highest level of *Hsp70* message after 30 and 60 minutes, but was not significantly different from the 6-copy genotype at these time points (Tables 2 and 3).

**Table 3:** **Relative fold increase in *Hsp70 *mRNA after 36°C exposure varies among copy number genotypes**

	**Duration at 36°C**		
***Hsp70 *genotype**	**15 min**	**30 min**	**60 min**
3 copy	123^1^	277	247
6 copy	331	428	390
12 copy	298	583	549

^1^ Fold increase is relative to control larvae (0 min at 36°C) of the same genotype and is corrected for the reference gene *Act5C* as described in the methods.

### Decreased recovery of metabolic rate after 36°C exposure

Cells metabolize energy stores to maintain ATP pools through increased respiration. We hypothesized that the rise in metabolic rate that we observed during the initial phase of the larval heat shock response at 36°C might reflect an increased use of energy stores and that this extra energy expenditure might have lasting effects on metabolic rate. We calculated the mean larval metabolic rate for 20-minute intervals after larvae returned to 22°C from 36°C. Table 1 and Figure 2B present these post-36°C metabolic rates as a percentage of the initial 22°C metabolic rate. Regardless of the duration of recovery, larvae of all genotypes had significantly lower 22°C metabolic rates post-36°C exposure when compared to their initial 22°C RMR (paired t-test for initial vs. 0-20 min recovery, *P=*0.001, <0.0001, <0.0001 for 3-, 6- and 12-copy genotypes, respectively; for initial vs. 95-115 min recovery, *P=*0.027, 0.008, 0.0006) (Figure 2B). The metabolic rates of all genotypes increased marginally during recovery time but were still significantly lower than their initial 22°C RMR at the end of the respirometry experiment (Table 1). The magnitude of this depression in metabolic rate becomes larger and more significant with increasing copy number (Figure 2B). The rank order of genotypes is the same across the entire duration of recovery (Table 1), although we did not have the power to detect significant differences between genotypes. The failure to recover metabolic rate could result from a genotype-specific decrease either in metabolic rate or in mass after 36°C exposure. We did not measure larval mass at the end of the respirometry experiments, because we wanted to allow larvae to develop and go through metamorphosis to ensure that the larvae we measured survived the protocol. However, larvae had access to food for the duration of the three-hour respirometry experiment and this is a time in development when larvae are typically gaining mass [38].

## Discussion

Our data reveal a rapid and transient increase in metabolic rate above the routine metabolic rate when *D. melanogaster* larvae are placed at 36°C, a temperature that induces a robust heat-shock response, and the magnitude of this increase is positively correlated with the number of *Hsp70* gene copies. Our results indicate that increased copy number affects *Hsp70* transcript abundance as early as 15 minutes after exposure to 36°C. Increased *Hsp70* copy number has been documented to increase Hsp70 transcript and protein abundance across different life stages in *D. melanogaster*[6,29,31], with 12-copy larvae expressing significantly higher levels of protein as early as 30 minutes after a one hour exposure to 36°C [6]. The dosage effect of *Hsp70* gene copy number on the rapid accumulation of both transcript and protein suggests that the genotypic effects that we detected on metabolic rate are associated with the energetic costs of inducible expression of this locus.

Increased expression of a protein-coding locus via transcription and translation requires multiple energy-dependent steps. Induction of gene expression requires the ATP-dependent processes of chromatin remodeling and the release of paused polymerase [22]. Nucleosome remodeling may be a particularly expensive component of gene expression, as the sensitivity of global transcription rates to ATP levels is alleviated when chromatin is experimentally de-condensed in cells [39]. In addition, there are energetic costs of nucleotide polymerization by *PolII* during transcription [22]. Translation requires additional energy input, and protein synthesis rates can depend on ATP concentration [11,40]. Finally, proteins often have their own energetic costs, such as the ATP-dependent function of the Hsp70 chaperone [41].

In wild type *D. melanogaster*, *Hsp70* exists in five to six copies, spanning two large genomic regions that are rapidly depleted of nucleosomes by an ATP-dependent nucleosome-remodeling complex within 120 seconds of external stimulus and are maintained in this state for at least 20 min of 36°C exposure [21]. Additional higher-order structural changes at these loci lead to maximal polytene-chromosome puff formation, indicative of decondensed chromatin, after 20-30 minutes of 37°C exposure in larvae [21,42]. During this time, the remainder of the genome acquires the mark of transcriptional quiescence [23], and translation of messages other than those of the heat shock proteins is dramatically reduced [19,20]. This presumably allows the diversion of resources towards the rapid and massive induction of *Hsp70*[19,20]. The differential rise in metabolic rate that we observe among copy number genotypes occurs soon after exposure to 36°C, during a period of differential *Hsp70* induction in the first 30 minutes of 36°C exposure. While all genotypes exhibit a rise in metabolic rate, 12-copy larvae increase their metabolic rate to a greater extent than either the 6- or 3-copy genotypes even though *Hsp70* mRNA levels of 6-copy larvae exceed that of 12-copy larvae at the earliest timepoint that we measured, with 12-copy larvae reaching the highest maximum level of induction after 30 minutes. The earlier and greater rise in metabolic rate in 12-copy larvae, that must remodel a larger part of the genome containing the extra gene copies, suggests that changing the chromatin structure of the *Hsp70* loci may contribute to the energetic demand of this inducible response, in addition to the costs of accumulating greater amounts of *Hsp70* transcript. Hsp70 protein does not generally accumulate to detectable levels until after 30-45 min of 36°C exposure [4,6,20], and 12-copy larvae do not accumulate significantly more Hsp70 protein until 30 min after a 1-hour exposure to 36°C [6]. However, the dramatic shift in translation away from preexisting messages and towards heat shock synthesis does occur in the first 20 minutes of 36°C exposure [20]. Thus, energetic costs involved in chromatin remodeling and translational control, as well as the costs of making *Hsp70* mRNA and protein, may all contribute to the metabolic response that we observed. Further experiments are warranted to understand the contribution of different molecular processes to the energetic cost shown to accompany the induction of this locus.

The rise in metabolic rate during induction can persist for up to 15 minutes and represents an increase in metabolic rate that is 21%, 25%, and 35% of the routine 22°C metabolic rate of 3, 6 and 12 copy genotypes, respectively. Rather than doubling energetic expenditure, inducing twice as many copies of *Hsp70* amounts to an ~1.3-fold increase in the rise in metabolic rate to meet the energetic costs of inducing this locus. This is consistent with the “self-limiting” regulation of the heat shock response; the accumulation of functional Hsp70 protein inhibits further transcription and destabilizes *Hsp70* mRNA [19], such that a doubling of gene copy number at this locus may not lead to a doubling of expression or associated energetic costs. To put the magnitude of this metabolic response in context, the rise in metabolic rate – 21-35% above the 22°C metabolic rate and 13-24% above the 36°C metabolic rate – is greater than the costs of terrestrial locomotion; walking increases metabolic rate 5-10% in adult flies [43]. In contrast, the costs of limbless locomotion and flight are much higher and result in 7-10 fold increases in metabolic rate [44,45]. In mammals, the energetic cost of total protein synthesis is estimated to constitute 18% of resting metabolic rate [46]. If protein synthesis constitutes a similar percentage of metabolic rate in insects, then the additional energy expended by a 6-copy larva as it expresses *Hsp70* could transiently equal or exceed its energy budget for protein synthesis.

In this context, the energy expenditure of inducing *Hsp70* may be large enough to create a transient deficit in the organismal energy budget. In fact, it takes 4-8 hours for adult *D. melanogaster* to regain metabolite homeostasis after inducing the heat shock response, with levels of glycogen, glucose and fatty acids reduced for 0-2 hours following 60 minutes at 36°C [47]; coincident with the decreased recovery of metabolic rate that we observed in larvae. If energy stores become reduced during the heat shock response, organisms may experience a dampening of glycolysis and aerobic respiration until energy stores are recovered to enable replenishing of the ATP pool. We observed the greatest depression of metabolic rate following 36°C exposure in 12-copy larvae, and larvae inducing 12 copies of *Hsp70* have been shown to have decreased locomotion post-induction [48]. These observations suggest that there are immediate costs to inducing the heat-shock response, and that these costs are higher for *D. melanogaster* expressing additional copies of *Hsp70*. These immediate, but transient costs of the heat-shock response may explain why the full benefits of the response are delayed in time relative to the accumulation of heat shock proteins [6,49]; 30 minutes after a 36°C exposure, 12-copy larvae have higher Hsp70 protein levels, but lower thermotolerance than do 6-copy larvae, and only achieve the benefit of an increased heat-shock response 60 minutes post-induction [6].

Tradeoffs between the costs and benefits of an increased heat-shock response in *Drosophila* have been extensively studied. Naturally occurring *Hsp70* gene duplicates experience extensive gene conversion, indicating selective pressure to maintain duplicate function [7], and increases in *Hsp70* gene copy number in *D. melanogaster* can confer increased thermotolerance [6,29]. However, excessive expression of *Hsp70* can reduce cell proliferation [50], delay larval developmental [51], depress larval locomotion after induction [48], and reduce adult fecundity [52]. Many of these costs of increased *Hsp70* expression have been attributed to chaperone-specific function [52]. However, our results show that changes in *Hsp70* gene copy number are also correlated with a dynamic alteration of metabolic rate, indicating that there are also energetic costs of inducing more copies. Thus, while extra genomic copies of *Hsp70* can confer increased thermotolerance, they do so at the price of increased energy expenditure. At least two additional lines of evidence suggest that such tradeoffs may underlie different evolutionary solutions to tolerate thermal stress. First, *D. melanogaster* adults from sub-equatorial Africa have low *Hsp70*-inducibility despite high thermotolerance, suggesting that alternative and potentially less-costly mechanisms of thermotolerance may evolve when individuals are chronically exposed to high temperatures [53]. Second, suppression of *Hsp70-*inducibility has occurred in both natural and laboratory populations of *D. melanogaster* via regulatory mutations that decrease inducible expression of *Hsp70* and that may have been selectively favored to reduce the energetic costs of living in environments that chronically induce *Hsp70* expression [7]. In fact, inducing *Hsp70* expression reduces thermotolerance in populations of *D. melanogaster* artificially selected for increased adult thermotolerance [54]. Presumably, stabilizing selection optimizes copy number at the *Hsp70* locus in *D. melanogaster,* balancing the advantage of additional copies that enable a more rapid or robust response to environmental stress with the disadvantage of physiological costs of expressing additional copies. In some environments, the costs of increased gene expression will outweigh the benefits, and in these environments, selection should favor the loss or silencing of duplicate gene copies.

Changes in gene expression, via gene duplication or regulatory change, represent a major class of adaptive mutations [55,56] and gene duplications have been an important source of adaptive mutations in *Drosophila*[57]. Yet, the overall role of selection in the evolution of gene duplicates remains debated [58], and purifying selection against mutations that affect gene copy number in *D. melanogaster* appears to be strong [59]. The fate of mutations that increase copy number likely depends on complex, locus-specific relationships between copy number, gene expression, protein abundance and fitness. There is a tendency for copy number to vary positively with expression level [60,61], and these types of mutations should require additional resource allocation to transcription, translation and downstream costs of protein function [12,13]. Our data reveal that a doubling of gene copy number at an inducible locus can generate detectable increases in the energy expenditure of a multicellular organism. How these energetic costs generate lifetime fitness costs is unknown, but will likely depend on the dynamics of gene expression at the locus (e.g. inducible versus constitutive expression). However, when the energetic costs of increased gene expression add to antagonistic-pleiotropic consequences for fitness, such as those discussed for the *Hsp70* locus, this will likely place constraints on the contribution of gene duplicates to adaptive phenotypic evolution.

## Conclusions

We have shown an energetic cost at the level of whole-organism metabolic rate associated with an inducible response to the environment. The magnitude of this energetic cost correlates positively with differences in *Hsp70* gene copy number and is on the order of other costs in the organismal energy budget, providing insight into the physiological and genetic mechanisms that potentially constrain the maintenance of gene duplicates and phenotypic evolution via increased gene expression. The evolution of inducible stress-responses is particularly impressive in the face of the energetic costs associated with increased gene expression.

## Abbreviations

ANOVA: Analysis of variance; ANCOVA: Analysis of covariance; ATP: Adenosine triphosphate; Hsp: Heat-shock protein; PCR: Polymerase chain reaction; qRT-PCR: Quantitative real-time polymerase chain reaction; RMR: Routine metabolic rate.

## Competing interests

The authors declare that they have no competing interests.

## Authors’ contributions

LAH and KLM conceived and designed the study. LAH conducted quantitative and standard PCRs, conducted respirometry experiments, conducted statistical analyses and wrote the manuscript. KLM assisted with statistical analyses and writing of the manuscript. Both authors read and approved the submitted manuscript.

## Supplementary Material

Additional file 1: Figure S1 Survival-to-adult to eclosion after 1 hour at 36°C is high and does not differ among *Hsp70* genotypes. Click here for file

Additional file 2: Table S1 Non mass-corrected metabolic rates of *Hsp70* copy number genotypes. Click here for file

Additional file 3: Table S2 Analysis of variance of *Hsp70* ΔCt values among copy number genotypes. Click here for file

Additional file 4: Table S3 Relative fold increases in *Hsp70* mRNA using an alternative calculation reveal the same pattern among copy number genotypes. Click here for file
