# Supplementary material for: Inducing extra copies of the Hsp70 gene in Drosophila melanogaster increases energetic demand
Source: BMC Evol Biol. 2013 Mar 19;13:68. doi: 10.1186/1471-2148-13-68 (PMC3641968; doi:10.1186/1471-2148-13-68)
Supplement: Additional file 2: Table S1 — Non mass-corrected metabolic rates of Hsp70 copy number genotypes. [file 1471-2148-13-68-S2.pdf]

Supplemental Table 1. Non mass-corrected metabolic rates of *Hsp70* copy number genotypes

	Hsp70 copy number genotype		
Metabolic trait	3 copy	6 copy	12 copy
22°C RMR[1]	25.49 ± 0.9 (a)[2]	27.28 ± 1.2 (a)	25.24 ± 1.0 (a)
36°C RMR	40.71 ± 1.2 (a)	43.17 ± 2.1 (a)	37.63 ± 1.2 (a)
36°C maxMR	45.85 ± 1.2 (a)	49.85 ± 2.2 (a)	46.82 ± 1.4 (a)

[1] Units for all measures other than percentages are μL CO_2/hr ± S.E. for 5 larvae from 23 replicates per genotype.

[2] Within each trait, different letters indicate significant differences between copy number genotypes ($P_{Tukey's}$<0.05).
