# Supplementary material for: Inducing extra copies of the Hsp70 gene in Drosophila melanogaster increases energetic demand
Source: BMC Evol Biol. 2013 Mar 19;13:68. doi: 10.1186/1471-2148-13-68 (PMC3641968; doi:10.1186/1471-2148-13-68)
Supplement: Additional file 3: Table S2 — Analysis of variance of Hsp70 ΔCt values among copy number genotypes. [file 1471-2148-13-68-S3.pdf]

Supplemental Table 2. Analysis of variance of *Hsp70* ΔCt values among copy number genotypes

	Duration of exposure to 36°C			
Factor	0 min	15 min	30 min	60 min
Genotype	F = 0.75 ^{NS}	F = 29.0 ***	F = 24.6 ***	F = 15.9 ***
Genotype effects[1]:				
6 copy – 3 copy	0.85 ^{NS}	2.28 ***	1.58 ***	1.64 ***
12 copy – 3 copy	0.56 ^{NS}	1.90 ***	1.66 ***	1.77 ***
12 copy – 6 copy	-0.29 ^{NS}	-0.38 ^{NS}	0.08 ^{NS}	0.13 ^{NS}

[1] Estimates using Tukey's post-hoc contrasts; positive values indicate higher expression on the ΔCt scale.
* P<0.05, ** P<0.01, *** P<0.001
