# Supplementary material for: Inducing extra copies of the Hsp70 gene in Drosophila melanogaster increases energetic demand
Source: BMC Evol Biol. 2013 Mar 19;13:68. doi: 10.1186/1471-2148-13-68 (PMC3641968; doi:10.1186/1471-2148-13-68)
Supplement: Additional file 4: Table S3 — Relative fold increases in Hsp70 mRNA using an alternative calculation reveal the same pattern among copy number genotypes. [file 1471-2148-13-68-S4.pdf]

**Supplemental Table 3.** Relative fold increases in *Hsp70* mRNA using an alternative calculation reveal the same pattern among copy number genotypes.

| | Duration at 36°C | | |
|---|---|---|---|
| *Hsp70* genotype | 15 min | 30 min | 60 min |
| 3 copy | 227[1] | 526 | 480 |
| 6 copy | 610 | 870 | 830 |
| 12 copy | 571 | 1127 | 1111 |

[1] Calculated using the ΔΔCt method to control for the reference gene *Act5C* expression in each sample
